# Facilitators of Adherence to the Study Pill in the FEM-PrEP Clinical Trial

**DOI:** 10.1371/journal.pone.0125458

**Published:** 2015-04-13

**Authors:** Amy Corneli, Brian Perry, Kawango Agot, Khatija Ahmed, Fulufhelo Malamatsho, Lut Van Damme

**Affiliations:** 1 FHI 360, Durham, NC, United States of America; 2 Impact Research and Development Organization, Kisumu, Kenya; 3 Setshaba Research Centre, Soshanguve, South Africa; Centers for Disease Control and Prevention, UNITED STATES

## Abstract

**Introduction:**

FEM-PrEP did not demonstrate a reduction in HIV acquisition because of low study pill adherence. Yet, plasma and intracellular drug concentrations indicated that some participants had evidence of recent pill use. We conducted a follow-up study to identify, among other topics, participants’ reasons for taking the study pill.

**Methods:**

Qualitative, semi-structured interviews (SSIs) were conducted with 88 FEM-PrEP participants. Participants were purposefully selected based on their adherence drug concentrations collected during FEM-PrEP and placed into three adherence interview groups: “high,” “moderate,” and “none/scarce.” Participants in the high and moderate groups described reasons why they adhered most or some of the time, including factors that facilitated their adherence. Participants in all groups described what they believed made it possible for *other* FEM-PrEP participants to adhere. In addition, 224 FEM-PrEP participants reported on their reasons for taking the study pills through a quantitative, audio computer-assisted self-interview (ACASI). Thematic analysis and descriptive statistics were used to analyze the qualitative and quantitative data, respectively.

**Results:**

Five themes were identified from the SSIs as facilitating factors of adherence: 1) participants’ support for the research, 2) HIV risk reduction, 3) routine formation and use of tools, 4) adherence counseling, and 5) partner awareness and support. Participants described similar facilitators when they spoke about other participants’ adherence. Among the 172 participants who reported in ACASI that they had taken a study pill, wanting to help answer the research question was the most frequently stated reason for taking the pills (94%, n = 161). We also found evidence of preventive misconception.

**Conclusions:**

Adherence was facilitated by personal motivations, such as risk reduction and interest in the research outcome, and by adherence strategies consisting of external cues, reminders, and support. These findings can inform future HIV prevention clinical trials and the rollout of effective antiretroviral-based HIV prevention technologies for women.

## Introduction

Four randomized, double-blind, placebo-controlled clinical trials have demonstrated the efficacy of oral tenofovir disoproxil fumarate (TDF) or oral TDF combined with emtricitabine (FTC) as pre-exposure prophylaxis (PrEP) for the prevention of HIV [1–4]. Two PrEP trials conducted among women in sub-Saharan Africa — FEM-PrEP and MTN-003 [Vaginal and Oral Interventions to Control the Epidemic (VOICE)] — did not demonstrate a reduction in HIV acquisition with oral FTC/TDF (FEM-PrEP and VOICE) or oral TDF (VOICE) [5,6].

FEM-PrEP was conducted in Bondo, Kenya; Bloemfontein and Pretoria, South Africa; and Arusha, Tanzania [5] (ClinicalTrials.gov Identifier: NCT00625404). Overall adherence to the study pill was observed to be low. After trial closure, an analysis of concentrations of plasma tenofovir (TFV) and intracellular tenofovir diphosphate (TFV-DP) from specimens collected at each 4-week study visit among a randomized prospective sub-cohort of 150 FEM-PrEP participants demonstrated that 23% of participants rarely took FTC/TDF, if ever. Drug concentrations fluctuated over time for 60% of participants. Yet, recent pill use was evident among some participants in the sub-cohort. Fifty-five percent of participants in the sub-cohort had at least one visit interval consistent with good adherence (i.e., TFV in plasma exceeding 10 ng/mL and intracellular TFV-DP in upper layer packed cells exceeding 100,000 fmol/mL) and 12% had evidence of good adherence for the duration of their trial participation (although none of these participants reached 52 weeks of follow-up due to the early closure of the trial) [7].

Several PrEP trials have identified demographic characteristics and other participant-related factors that are associated with adherence, such as age, marital status, certain sexual behaviors, and perceived HIV risk [6–10]. To further the field’s understanding of facilitators to study product adherence in placebo-controlled PrEP clinical trials, participants’ own voices describing their reasons for taking the study pill — at least some of the time — should be heard. We conducted a follow-up study with former FEM-PrEP participants at two of the FEM-PrEP sites—Bondo, Kenya, and Pretoria, South Africa—to primarily identify participant-reported reasons for taking and not taking the study pill within the context of a placebo-controlled clinical trial. Here we describe factors that facilitated adherence. Findings on the reasons for non-adherence are described elsewhere [11]. Such data on participant-defined facilitators of adherence not only will inform future clinical trials of investigational antiretroviral (ARV)-based HIV prevention products but also can promote product use in the rollout of effective ARV-based HIV prevention technologies for women.

## Methods

### The FEM-PrEP Clinical Trial

The details of the FEM-PrEP clinical trial have been described elsewhere [5,7,12]. In brief, participants were asked to take their randomly assigned study pill—either FTC/TDF or placebo—daily for 52 weeks. Adherence counseling was provided by trained study counselors at each 4-week study visit [13]. Adherence support tools—pill boxes and calendars—were offered, and participants identified and refined (as needed) personalized adherence plans during each counseling visit, including specific strategies for integrating study pill use into their daily lives.

### Drug Concentrations

For the main FEM-PrEP adherence analyses, a semi-ordinal, composite adherence score was developed, in collaboration with the study’s pharmacologist, using plasma TFV and intracellular TFV-DP concentrations from blood specimens collected at every 4-week study visit [7]. Participants were assigned a composite adherence score for every study visit interval in which blood specimens were available. Scores ranged from 0 (concentrations consistent with low or no doses of drug) to 5 (concentrations consistent with taking the study drug nearly every day) (Table 1). A maximum of 13 visit intervals were possible.

**Table 1:** Qualitative adherence composite scores, corresponding TFV and TFV-DP concentrations, and estimated doses per interval [7].

Adherence composite score	TFV in plasma and TFV-DP in upper layer packed cells	Estimated doses per interval
0	No detectable TFV and <10,000 fmol/mL TFV-DP	A low number of doses or no doses at all in the interval
1	Detectable TFV but <10,000 fmol/mL TFV-DP	A few doses in the entire interval
2	10,000–100,000 fmol/mL TFV-DP, regardless of TFV	1–2 doses per week
3	<10 ng/ml TFV and >100,000 fmol/mL TFV-DP	Several doses early in the interval, followed by a stop in the week or two leading up to the sampling visit
4	>10 ng/ml TFV and 100,000–1,000,000 fmol/mLTFV-DP	4–5 doses per week
5	>10 ng/ml TFV and >1,000,000 fmol/mL TFV-DP	Approximately daily dosing

### Sample Selection and Data Collection

As part of a larger follow-up study to FEM-PrEP, we conducted semi-structured interviews (SSIs) on adherence with former FEM-PrEP participants who were assigned FTC/TDF during the trial. We chose to conduct the follow-up study in the Bondo, Kenya, and Pretoria, South Africa sites because they had qualitative research previously embedded within the FEM-PrEP clinical trial, they had the most participants enrolled in FEM-PrEP, and they were the sites with the highest number of participants who seroconverted. As described in more detail elsewhere [14], participants were selected from a list of former FEM-PrEP participants who had previously given permission to be contacted for future research or through community-wide promotion of the study; all had previously had their blood specimens analyzed for drug concentrations as part of other FEM-PrEP analyses.

Based on their adherence drug concentrations, we placed the participants into three interview groups: “high,” “moderate,” and “none/scarce.” We did not require a minimum number of specimens for inclusion in a group. Rather, we chose to consider all available specimens (up to 13 per participant) when deciding in which adherence group to place participants, as we believed this would provide for a richer discussion of adherence. Because of the early closure of the trial, however, many participants were unable to complete 52 weeks on the study drug and therefore had fewer specimens available for testing of drug concentrations. Moreover, a composite adherence score was not calculated for a study visit interval when a participant missed a study visit, when the participant did not have study pills available to use (e.g., because of a protocol-defined product withdrawal or a missed pill supply visit), or when there was no or insufficient specimen for analysis [7]. Consequently, some participants had composite adherence scores for all 13 study visit intervals, but many had fewer scores available. We therefore categorized participants based on a combination of the composite adherence score for each study visit interval and the number of available specimens.

Participants who had composite adherence scores of 4 or 5 for the majority, if not all, of their available samples were identified for recruitment into the high adherence group. For the moderate adherence group, we identified participants for recruitment who had composite adherence scores that fluctuated over time among the available samples, remained steady between scores 2 and 4, or were higher at the beginning of the trial and lower as time passed. Participants were identified for the none/scarce group if the majority of their recorded measurements had scores of 0. We ranked the order in which to recruit women based on the number of specimens available (i.e., women who had a higher number of recorded specimen measurements were placed higher on the recruitment list for each group). We aimed to interview approximately 15 participants per group, per site.

**Fig 1:**
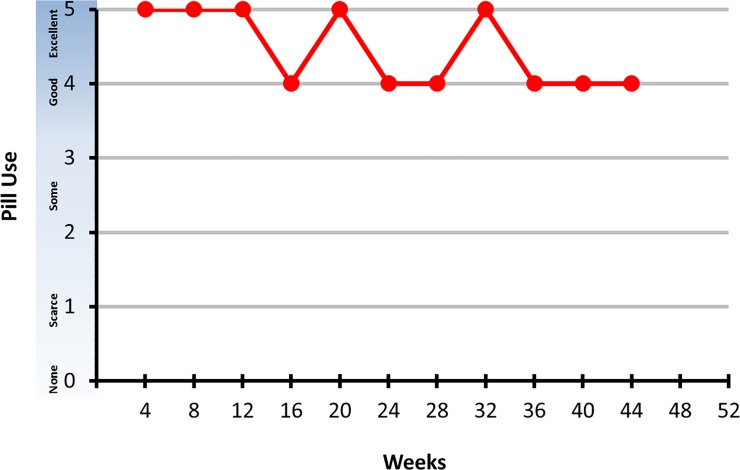
Example of adherence composite score graph shown to participants.

During the SSIs, participants viewed a graph displaying their composite adherence scores over the 13 study visit intervals as a discussion aid (Fig 1). Only participants in the high and moderate groups were asked to describe reasons why they adhered to the study pill regimen. We first asked a broad question to participants in the high adherence group: “What made it easy for you to take the study pills?” After responding to the question, participants were asked direct probes about other potential reasons, to explore whether any of those other factors contributed to their adherence. The probes included perceived HIV risk, belief that the pill was efficacious for HIV prevention, and partner support. Before moving to the next section, participants were asked an indirect probe to identify any other possible reasons for taking the study pill.

Participants in the moderate group were asked about what led them to take the study pill some of the time but not all of the time and about times during the trial when they took the study pill more often. Follow-up probes were asked that focused on potential events or experiences that might have motivated adherence, such as engaging in perceived risky sexual behavior, being unsure of partners’ HIV status, and receiving adherence counseling; an indirect probe was also asked to explore other possible reasons.

Personalized questions were asked to participants in both the high and moderate group when appropriate (e.g., the participant had fluctuating adherence composite scores) to specifically probe about the context surrounding the participant’s individual adherence patterns. Participants in the none/scarce group were not asked questions on reasons they may have taken the study pill at times. Instead, they were asked to describe what they believed made it possible for *other* FEM-PrEP participants to take the study pill some or most of the time, as well as about times during the trial when other participants were able to take the study pill more often. Participants in the moderate and high groups were also asked these questions.

A quantitative, audio computer-assisted self-interview (ACASI) was offered to all former FEM-PrEP participants who participated in the larger follow-up study. ACASI was completed by 1) participants from the three SSI adherence groups described above (i.e., high, moderate, and none/scarce), 2) participants assigned placebo during FEM-PrEP, and 3) former FEM-PrEP participants who participated in a separate SSI on risk perceptions. Details regarding the eligibility criteria and recruitment of these populations have been described elsewhere [14]. We chose to gather data using ACASI, in addition to SSIs, because ACASI can potentially reduce socially desirable responses [15] and because previous FEM-PrEP findings showed that participants over-reported their adherence in the interviewer-administered quantitative surveys and SSIs conducted during the clinical trial [12]. All participants were informed that their ACASI answers would not be linked to them or to their demographic information, to further reduce the likelihood of social-desirability bias. During ACASI, participants were asked to think about the study pills that they took during the trial. They were then asked to provide a yes or no response to six possible reasons for taking the study pill. Participants were also asked to report if they had never taken a study pill.

### Data Analysis

#### Qualitative data analysis

Applied thematic analysis was used to analyze the qualitative data on the reasons for adherence [16]. The SSIs were audio-recorded and simultaneously translated and transcribed into English using a standardized transcription protocol [17]. Four analysts structurally coded the transcripts using NVivo 10 [18] to segment text on self-reported reasons for taking the study pill and perceived reasons other participants took the study pill. Inter-coder reliability was routinely assessed throughout the coding process, but more heavily at the beginning to ensure a common understanding of the codebook and interpretation of the data. Ten percent of the transcripts were selected to be individually coded by each of the analysts to assess reliability. At each scheduled assessment of inter-coder reliability, discrepancies in the application of codes were identified and resolved, and transcripts were recoded as needed. Once structural coding was complete, coding reports were produced for each structural code.

Coding reports were then analyzed by a single analyst for emergent content-driven themes and sub-themes. Thematic coding was done through an iterative and inductive process of identifying and defining new themes as they emerged in the participants’ narratives, coding the text, and then re-analyzing previously coded text for similar themes. Once all relevant content was fully captured, the primary analyst prepared a written summary of the main findings, and another analyst independently reviewed the coding reports to verify each theme and sub-theme described in the summary. Any discrepancies in interpretation of the data or frequency of themes and sub-themes were discussed until agreement was reached.

Based on one of the themes in the data — HIV risk reduction — we reviewed participants’ narratives for specific *a priori* concepts on preventive misconception [19], including logical preventive misconception [20], and deductively indexed text related to these concepts. Simon and colleagues [19] define preventive misconception as “the overestimate in probability or level of personal protection that is afforded by being enrolled in a trial of a preventive intervention.” Woodsong and colleagues [20] have expanded the concept, adding that some participants may hold a “logical preventive misconception,” which is “participants’ articulation that if the trial drug is proven effective, they will have been protected while they were in the trial.” We identified salient texts from the transcripts and evaluated them to determine whether there was evidence of preventive misconception, using both definitions described above. Text suggesting such evidence was further analyzed to categorize participants’ rationales for their beliefs into sub-themes: preventive misconception, logical misconception, or emergent themes. A second analyst independently reviewed the text to identify whether it showed evidence of preventive misconception and how it should be categorized. Discrepancies were discussed until final agreement on the interpretation of the data was reached.

#### Quantitative data analysis

Response frequencies were calculated for the ACASI data. Because we focused on reasons for taking the study pill, participants who indicated that they had never taken a study pill were removed from the analysis.

### Ethics Statement

The Ethics Review Committee (ERC) at the Kenya Medical Research Institute (KEMRI) (Bondo), the Pharma-Ethics Review Board (Pretoria), and the Protection of Human Subjects Committee (PHSC) at FHI 360 in the United States reviewed and approved the research. Participants provided either verbal (Bondo) or written (Pretoria) informed consent to participate in the follow-up study. The use of verbal consent was requested and approved by the KEMRI ERC and the PHSC for the Bondo site because the research was considered minimal risk and verbal consent has been previously approved for social-behavioral research in Kenya. Study staff signed and dated an information sheet documenting that verbal consent was indeed provided by each individual; the participants’ identification number for the follow-up study was listed on the sheet. We sought and received approval by the Pharma-Ethics Review Board and the PHSC for written consent for the Pretoria site based on local guidelines that required written consent. The written consent form included the same information that was included on the verbal consent information sheet; the only difference between the two documents was the signature line.

## Results

### Study Participants and Adherence Scores

**Fig 2:**
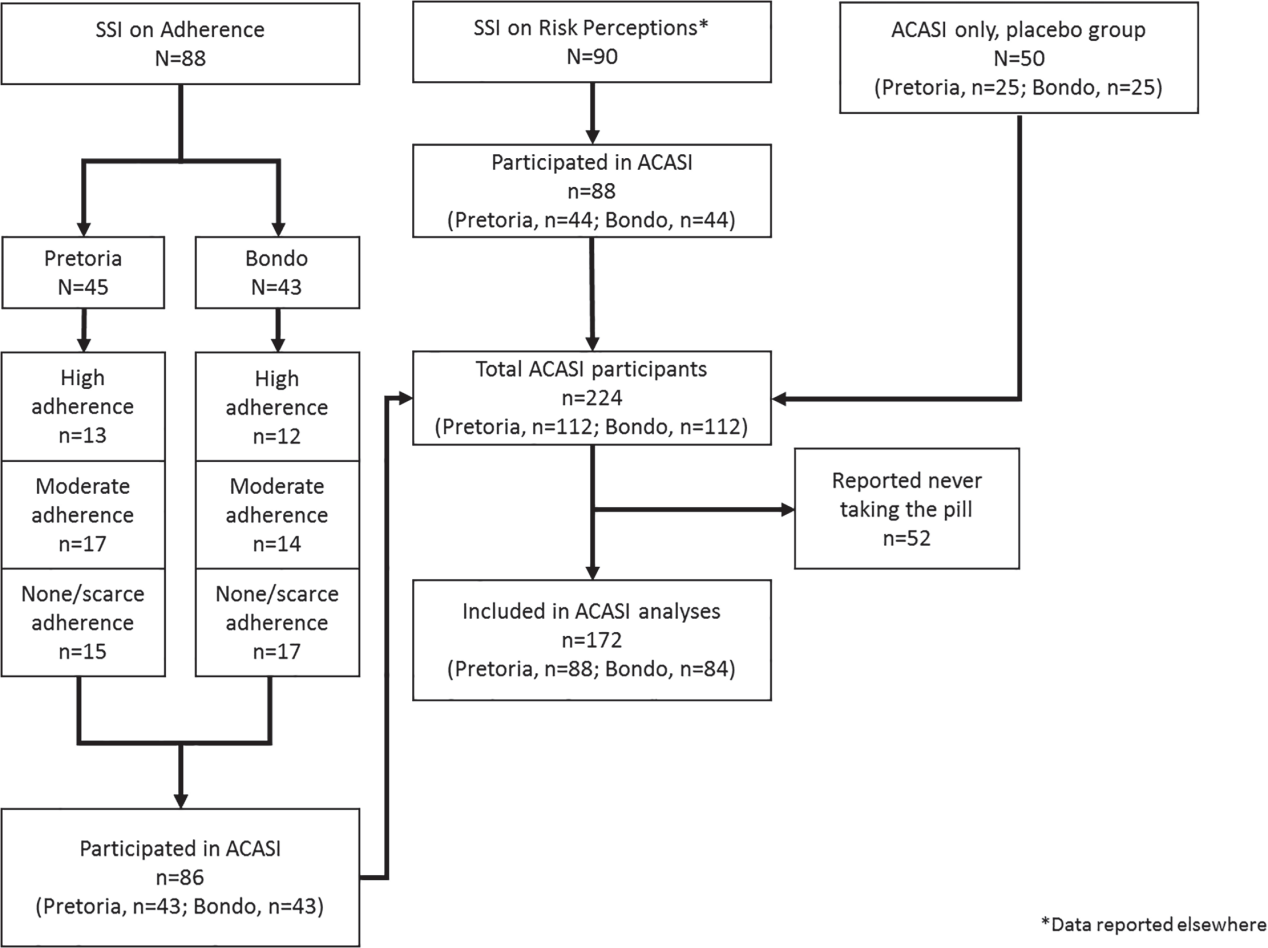
Sample size.

Fig 2 displays the total number of participants who took part in the SSIs and ACASI. For the SSIs, we interviewed 88 women in the high (n = 25), moderate (n = 31), and none/scarce (n = 32) adherence groups. Among participants in the high adherence group, the median number of specimens was six (range: 1 to 13) and the composite adherence scores ranged from 2 to 5. For 84% of participants in this group, the mean composite adherence score for all visits ranged from 4.0 to 5.0. The four participants whose mean score was below 4.0 (i.e., between 3.6 and 3.9) had between 11 and 13 samples, and seven to 10 of those samples had scores of 4 and 5.

For participants in the moderate group, the median number of specimens was five (range: 2 to 13) and the composite adherence scores ranged from 0 to 5. Participants’ mean composite adherence scores for all their visits ranged from 1.0 to 3.8. The three participants whose mean scores overlapped with those of participants in the high group (i.e., between 3.7 and 3.8) each had fewer available specimens (three to five) than did participants in the high group, and several of their composite adherence scores were under 4.

ACASI was completed by 224 participants. However, we excluded responses from 52 participants (23%) who reported that they never took a study pill, leaving a sample size of 172 participants.

### Semi-Structured Interviews: Participants’ Own Reasons for Taking the Study Pill

Participants’ description of the reasons why they took the study pill focused on two kinds of facilitators: motivating factors and adherence strategies. We grouped these facilitators into five themes: 1) participants’ support for the research, 2) HIV risk reduction, 3) routine formation and use of tools, 4) adherence counseling, and 5) partner awareness and support.

#### Participants’ support for the research

Fourteen participants in the high adherence group and four in the moderate group explained that they were motivated to take the study pill because they supported the research. Half of these narratives (n = 9) illustrated the participants’ strong interest in learning whether FTC/TDF was effective in preventing the acquisition of HIV:

*I wanted to know the truth about those drugs and that is the reason why I took them daily*, *if they could work*. *Even though I did not know the drug I was using*, *I just wanted to know if it worked or not*. *(Bondo*, *high group)*

*I wanted to see how the results will go at Setshaba [i*.*e*., *FEM-PrEP site] when I drink the pills*. *(Pretoria*, *high group)*

Narratives from the other nine participants included statements that are characteristic of altruism. Participants specifically described being motivated to take the study pill so that the results of the study could help others, their children, or future generations:

*I was taking it because I wanted [to know] if could be found to prevent—if it [can be used to] prevent [HIV for] the generation that is behind us*. *(Bondo*, *high group)*

*The reason I took it more often was because*, *just in case the study result comes out positively*, *it can help some other people who were not in the study*. *So that it can help others in the future*.

*(Bondo*, *moderate group)*

Some of these altruistic narratives suggested a sense of the participants' commitment toward the research process and toward their partnership with the research team:

*The reason I took it was that I wanted to see if it really works or not*, *this pill*. *I wanted them to get the results as to whether it works or not*. *(Pretoria*, *high group)*

*I took it because I wanted the results to come out correctly*. *Because if we do not take it*, *then we are wasting our time by going there*. *(Bondo*, *high group)*

#### HIV risk reduction

Twelve participants in the high adherence group and 18 in the moderate group said they were motivated to take the study pill because they believed they were at risk of HIV or because they thought the study pill might reduce their risk. Many of these participants (n = 18) described that their sexual partner—generally their primary partner—might or did have other sexual partners; they described lacking trust in their partners’ ability to remain HIV-negative:

*It is because I wanted to protect myself*. *I can’t trust my boyfriend because he stays there and I am staying here*. *I can’t see everything that he does*. *Even if he can use a condom*, *it is possible that it can break*. *It is also possible that he can forget to use a condom with another girl*, *like when he drinks alcohol*. *So*, *I can’t say I trust him*. *(Pretoria*, *high group)*

A few participants (n = 3) said their partners’ HIV-positive status motivated them to take the study pills. Several others (n = 8) described being motivated to adhere because they were unaware of their partners’ HIV status:

*I think that*, *because according to the person [i*.*e*., *casual partner]*, *I did not know his status*, *when I thought of that*, *I was really taking the drug how it was required*. *(Bondo*, *moderate group)*

Narratives from a few participants (n = 3) focused on being motivated to take the study pills because of their own high-risk behavior of having multiple sexual partners:

*In my mind*, *I knew that I could get HIV at any time and that is the reason for me taking it [i*.*e*., *the study pill]*. *[Interviewer question*: *Why do you say that you could get it at any time*?*] As long as you have a “mpango wa kando” [Interviewer note*: *This means an extra sexual partner]*, *that is it*. *And*, *also being that I am here and my husband works outside*, *I may not know how he is*, *and those are what made it easy for me [to take the study pill]*. *(Bondo*, *high group)*

Participants’ narratives about being motivated to adhere to the study pill so that they could reduce their HIV risk, as well as other similar statements made in the interviews (such as reasons for joining FEM-PrEP), suggested that some participants may have had preventive misconceptions about the study pill. After further analysis of the data, we found evidence that all participants had a general understanding of the purpose of the trial; all but five described that participants could have been assigned either FTC/TDF or placebo. (The remaining five participants were not asked questions about the placebo, but all described that the trial was testing FTC/TDF for HIV prevention). Yet, narratives from seven participants (58%) in the high group and six (33%) in the moderate group who said they adhered to the study pill to reduce their HIV risk provided evidence, or probable evidence, of preventive misconception. Statements from these participants suggested that they believed the study pill would protect them from HIV, although their reasons for these beliefs varied. A few participants in the moderate group (n = 3) simply stated that they were motivated to adhere to the study pill so they could be protected from HIV; no or few additional explanations were given:

*That’s what made me happy…I was tested when I went there [i*.*e*., *FEM-PrEP clinic]*, *and given drugs so that they protect me*. *[Interviewer question*: *Protect you to do what*?*] So that I don’t get infected with HIV/AIDS*. *(Bondo*, *moderate group)*

For others (n = 10), however, their rationales for feeling protected from HIV and for believing—or hoping—they were assigned FTC/TDF were more nuanced. Explanations from some participants (n = 4) were grounded in the belief that *if* FTC/TDF is proven effective for HIV prevention, they would have been protected:

*I wanted to see the results*…*because I knew that it was going to work*. *And who knows that I might just be one of the victims who [might] get sick [i*.*e*., *HIV]*. *And if it could prevent*, *maybe I would be the first person to see that*. *(Pretoria*, *high group)*

Some participants’ narratives (n = 6) described that certain events, such as having multiple HIV-negative tests (n = 3) or side effects (n = 1), were evidence that they were assigned FTC/TDF. For example, one participant who adhered well described a combination of hope and perceived evidence as motivation:

*I was taking it because I had hoped that sometimes it could protect me because you know some time back my husband was found to be HIV-positive though he did not tell me…Because when I heard that it could protect me from getting HIV I got encouraged*…*I was taking this pill*. *I was protected*. *I did not get HIV*. *That is why it was easy for me to take my pills daily*. *I was also tested every month and I knew my HIV status and so this encouraged me more to take my pills daily*. *I knew it was working because I did not ever get HIV from my husband*. *(Bondo*, *high group)*

Similarly, two of these participants described that over time they came to believe they were assigned FTC/TDF and it was working. However, neither participant provided an explanation (Note: participants referred to FTC/TDF by its trade name Truvada.):

*So we joined because we knew we had other sexual partners and we wanted to see if this drug could work in protecting us from getting HIV*. *And so we used this pill and by the time we were six months in the study*, *we knew we were taking Truvada and it is preventing HIV infection*. *(Bondo*, *moderate group)*

#### Adherence counseling

Five participants in the high adherence group and 12 in the moderate group spoke about their motivation to adhere after adherence counseling. Seven of these participants simply gave general, non-descriptive “encouragement” statements about their motivation to adhere after participating in adherence counseling. The narratives of the remaining participants did not focus on the helpfulness of the pill-taking strategies discussed in adherence counseling, such as brainstorming about potential strategies to overcome any barriers they may have faced in taking the pill. Rather, they focused on two factors that were strengthened by adherence counseling: 1) a sense of reassurance that staff can help them in case they experience any side effects (n = 5) and 2) their dedication to contribute to answering the research question (n = 6):

*Mostly if we go there the [counselor] talks to you*. *I feel that I regain my heart [Interviewer note*: *getting motivated] because how they put it to test to know if that thing works*. *(Bondo*, *moderate group)*

#### Routine formation and use of tools

Twelve participants in the high adherence group and 13 in the moderate group explained that they remembered to take the study pill because they integrated daily pill taking into their everyday lives by establishing a routine, they used adherence reminder tools, or they became “used to” taking the pills regularly. Establishing a daily time to take the study pill—usually linked to another activity such as going to bed, waking up in the morning, or doing chores—was the most common adherence strategy described for remembering to take the study pill (n = 17):

*So for me [at the beginning of the study when I took the pills]*, *the time I chose really helped me because I had mastered it*. *I could wake up at 5 a*.*m*. *and take it*. *That is when I could start doing my chores*. *(Bondo*, *moderate group)*

Setting a reminder alarm, with or without the use of other strategies, was also frequently mentioned (n = 8):

*I used to set [my phone alarm] at 8*:*00 every day*. *Like I never forgot*. *At night*. *Even if the phone rings and maybe I don’t hear it*, *I know that when it’s time for Generations [Interviewer note*: *A soap opera on weekday nights]*. *I know it’s my time to drink pills*. *Then I fetch water*, *sit down and drink*. *(Pretoria*, *high group)*

Several participants (n = 6) also spoke about keeping the pills with them or keeping them visible, such as on a table near their bed, so they could see them every day:

*I kept those pills where I could see them*. *Now anytime I was ready to go to sleep*, *I could see them and then I remembered*. *Then I did what*? *I took them*. *Anytime I was going to sleep I could see them*, *because I was taking them when I [was] going to sleep*. *(Bondo*, *high group)*

Only four participants mentioned that using the study-provided pill box (n = 3) or calendar (n = 1) helped them to adhere.

#### Partner awareness and support

Narratives from 19 participants in the high adherence group and eight in the moderate group illustrated a wide range of partner involvement, from no partner engagement or knowledge of trial participation to active adherence support. Several participants (n = 8) described receiving encouraging and helpful support from their partners. These narratives focused primarily on situations in which the partner regularly reminded the participant to take her study pill:

*He would remind me that*, *“It is 10*:*00*. *Have you taken it*?*” After he had reminded me*, *I would take it*. *(Bondo*, *high group)*

*My husband would tell me to drink them so that they can find out the results*, *you see*? *Just that me*, *when he wasn’t here*, *I didn’t drink them*. *I was lazy to fetch them*. *(Pretoria*, *high group)*

More participants (n = 14), however, described situations in which they disclosed their FEM-PrEP participation to their partners and their partners merely acquiesced to their pill use. In these situations, participants described that their partners’ knowledge of their trial participation helped them to adhere, simply because their partner did not discourage or interfere with taking the study pill. These participants said that they did not rely on their partners for adherence encouragement and support but described or implied that their partners’ awareness made it easier for them to adhere because their partners did not give them “any problems”:

*I gained courage and shared with my partner*, *and I did not get a lot of problems with him*. *So*, *I found it easy to take my pills*. *(Bondo*, *high group)*

*Support*? *There was no support he was giving me at all*. *But the thing is he never stopped me*. *But*, *to remind me that today*, *“what about your pills*?*” That one was not in his mind at all*. *(Bondo*, *high group)*

A few participants (n = 5) in the high and moderate groups said they did not inform their partners of their participation in FEM-PrEP, primarily because they did not live with their partners or because their partners would not agree to their participation. Therefore, these participants did not describe receiving any assistance, support, or discouragement from their partners.

### Semi-Structured Interviews: Perceptions of Reasons for Other Participants’ Adherence

Participants described reasons similar to the themes above when they spoke about facilitators to adherence among other participants. One additional reason emerged that was rarely mentioned (n = 4) when participants described their own motivations: taking the study pill prior to a study visit to appear adherent. Although participants mentioned this reason only when talking about other participants who were inconsistent adherers (versus high adherers), 28 participants (23 were from Pretoria) said they believed participants took the study pill immediately before their next follow-up visit to ensure that the drug was in their blood sample.

### ACASI

In both sites, the most frequent reason given during ACASI for taking the study pill was to help answer the research question on whether FTC/TDF can prevent HIV (94%, n = 161) (Table 2). Many participants were also motivated to take the study pill because they believed it would provide protection against HIV (77%, n = 132) or because they believed that they were assigned FTC/TDF during the trial (61%, n = 105). Having a perception of high HIV risk also served as a motivating factor for many participants (56%, n = 97), although this factor was more common in Bondo (71%, n = 60) than in Pretoria (42%, n = 37).

**Table 2:** Reasons given during ACASI for taking the study pills in FEM-PrEP, by site, n (%).

Item	Bondo(n = 84)	Pretoria(n = 88)	Overall(n = 172)
To help answer question of can FTC/TDF prevent HIV	78 (93)	83 (94)	161 (94)
Thought pills would protect against HIV	66 (79)	66 (75)	132 (77)
Thought pills were FTC/TDF	47 (56)	58 (66)	105 (61)
Believed had a high chance of getting HIV	60 (71)	37 (42)	97 (56)
Thought they would treat an illness you had	21 (25)	20 (23)	41 (24)
Other participants were taking them	6 (7)	24 (27)	30 (17)

Overall, when responses from both sites were combined, few differences were found between the responses of participants assigned FTC/TDF and responses of participants assigned placebo (Table 3). Specifically, we did not see a difference in responses between the two groups regarding taking the study pill because they believed they were taking the active drug (59% of participants assigned FTC/TDF, n = 36; 58% of participants assigned placebo, n = 22). However, more participants assigned FTC/TDF (79%, n = 48) than assigned placebo (66%, n = 25) thought the study pill would protect them against HIV, although the difference was not significant (p = 0.17).

**Table 3:** Reasons given during ACASI for taking the study pills in FEM-PrEP, by study arm, n (%).

Item	TDF/FTC(n = 61)	Placebo(n = 38)
To help answer question of can FTC/TDF prevent HIV	58 (95)	35 (92)
Thought pills would protect against HIV	48 (79)	25 (66)
Thought pills were FTC/TDF	36 (59)	22 (58)
Believed had a high chance of getting HIV	34 (56)	17 (45)
Thought they would treat an illness you had	18 (30)	6 (16)
Other participants were taking them	11 (18)	7 (18)

## Discussion

We identified numerous facilitators that motivated or assisted participants to take the study pill, at least some of the time, during FEM-PrEP. Participants reported that they adhered primarily because of 1) personal motivations, which were HIV risk reduction and a general interest in the outcome of the research or altruism; and 2) adherence strategies, which consisted of external cues, reminders, and support, such as partner awareness, encouragement and support, or assistance; established routines and tools; and adherence counseling. Based on the findings described here, women may need several kinds of tools and approaches to facilitate their adherence to a daily pill for HIV prevention.

The findings on partner engagement suggest that partners who were aware of trial participation played a role in facilitating adherence in several different ways. First, some partners actively encouraged and supported trial participation. In response, participants may have been motivated to adhere to the study pills because they had the support of their partners. Second, and often combined with the active support described above, some partners provided tangible support by regularly reminding participants to take their study pill. Third, and quite different from the first two ways in which partners supported adherence, some partners did not object to trial participation, encourage it, or interfere with it. In this situation, participants may have used partner *awareness* as a strategy for removing a barrier to adherence. For example, disclosing trial participation to their partners may have alleviated the burden of keeping study participation a secret, or may have eased the anxiety of experiencing potential negative consequences if their partners learned of their study participation. Partner awareness may have also allowed participants to be more actively engaged in certain adherence strategies (e.g., leaving pill bottles on a bedroom table). Yet, active support from partners may not be necessary for some women to adhere. Partner awareness of trial participation alone may be all the support some women need from their partners to take a daily, study pill. Passive partner support was also described in a qualitative study conducted among participants from the Johannesburg site of the VOICE study; participants perceived that their partners passively approved of the use of the study product, as long as it did not interfere with the relationship [21].

Partner engagement in women’s use of ARV-based HIV prevention products has received considerable attention within oral PrEP and microbicide trials [22–28]. Recently, Lanham and colleagues [28] summarized partner engagement data from several microbicide clinical trials and reported a continuum of partner involvement—from opposition to agreement or non-interference to active support. Their findings are similar to the spectrum of partner support reported among FEM-PrEP participants, described here and elsewhere [11]. Among participants in the CAPRISA 004 microbicide clinical trial, a significant positive association was found between disclosure of trial participation to partners and adherence to the study gel, although the strength of the association was modest [24]. Data from these studies suggest that male engagement activities should be included in placebo-controlled clinical trials, as well as in the rollout of new HIV prevention products, to enhance male partners’ understanding, acceptance, and support of such methods. Ultimately, however, each woman should decide on the amount and type of partner support and awareness she wants and needs to adhere to products that reduce her risk of HIV [28] — within and outside of the context of a placebo-controlled clinical trial.

The study’s findings also suggest that perceiving one’s self to be at risk of HIV may encourage some participants to adhere to a study pill. Indeed, we have reported elsewhere the statistically significant association between having some perceived HIV risk and good adherence [10]. Similarly, some participants in the VOICE qualitative study in Johannesburg described enrolling and taking the study product because of a sense of risk [21]. In placebo-controlled HIV prevention trials, however, caution is needed, particularly when enrolling those who feel at risk of HIV, to ensure that participants are not motivated by misconceptions about the preventive effectiveness of the product under investigation [19,20]. During FEM-PrEP, participants were reminded at each study visit that they may have been assigned either FTC/TDF or placebo (and told that the placebo cannot protect against HIV), and that the purpose of the research was to determine whether FTC/TDF was effective for HIV prevention. They were also counseled to use HIV risk reduction methods of known effectiveness during the trial. However, even if regular reminders are provided about the investigational nature of the study product, misconceptions or misunderstandings can occur or emerge over time. Preventive misconception is a concern within placebo-controlled trials because it may demonstrate potential problems with the informed consent process or could lead to increased risk behaviors (e.g., using condoms less frequently) from believing that the investigational product will provide protection [19].

Although our study was not designed to investigate preventive misconception, thus making it difficult to fully discern and understand participants’ rationales for believing that the study pill would protect them from HIV, we did not see evidence of concerns related to informed consent or risk compensation in our data. However, it appears likely from the available data that some participants were motivated to take the study pill because they underestimated the likelihood of being assigned the placebo. Based on this underlying premise, some participants may have held “logical preventive misconceptions” [20] and believed they would have benefited (i.e., remained HIV-negative) during the trial if FTC/TDF was shown to be effective at the end of the trial; others may have held other beliefs that, from their perspectives, were rational conclusions that the study pill prevented HIV based on their experience of taking the study pill during the trial (e.g., they remained HIV-negative while at continued perceived or actual risk of infection). Participants’ narratives related to preventive misconceptions, however, may have been influenced by the timing of data collection, as we collected these data after the trial was over, when participants’ HIV statuses and pill assignments were known.

Preventive misconception has been identified in other ARV-based HIV prevention clinical trials. The term “logical misconception” emerged from a secondary analysis of data collected among participants in the HPTN 035 microbicide clinical trial; some participants also appeared to hold preventive misconceptions based on the original definition [20]. In the CAPRISA 004 microbicide clinical trial, researchers systematically evaluated preventive misconceptions among participants as they exited the study; findings demonstrated that 1) a small percentage of participants (15%) held preventive misconceptions about the study product, 2) there were no associations between such misconceptions and indicators related to overall trial misunderstanding, and 3) condom use was lower among participants who believed the study product protected them from HIV [29]. Future studies of ARV-based HIV prevention products should consider incorporating data collection on preventive misconception into their planned assessments [30].

Within FEM-PrEP and other placebo-controlled oral PrEP and microbicide clinical trials [13,31,32], substantial efforts were made to design supportive adherence interventions; the interventions implemented in Partners PrEP and CAPRISA 004 were effective at increasing adherence [31,33]. Even though adherence in FEM-PrEP was low overall, participants’ descriptions of adherence strategies suggests that establishing an adherence plan during adherence counseling, including reminder strategies and ways to incorporate pill taking into their everyday lives, may have helped some participants remember to take the study pill. Based on these findings, we recommend that PrEP adherence counselors explore with women the most appropriate external cues and reminders that will facilitate adherence. Nevertheless, for participants in the moderate adherence group, adherence counseling and other motivations described here appear to have been only marginally or temporarily effective at motivating these participants to adhere; in these cases, the barriers participants faced in FEM-PrEP [11] may have challenged their motivations and ability to adhere.

Women’s perceptions that many participants were taking the study pill immediately prior to a study visit in order to appear adherent were not supported by drug concentration data [7]. However, the misreporting of adherence was common in FEM-PrEP. We have described elsewhere that participants over-reported their adherence during the trial [12]; the primary reason for over-reporting was an incorrect assumption that they would be terminated from the trial for non-adherence [14].

Our study had several limitations. First, we did not design the study to compare reasons for adherence between the adherence groups; the ACASI data could not be aggregated by adherence group (due to procedures in place to reduce socially-desirable responses), and different questions and probes were asked to participants in each SSI adherence group based on factors we believed might be relevant to their specific levels of adherence. We were, however, able to identify the overall facilitators of adherence among participants who took the pill most or some of the time. From the SSI findings, these factors were similar between the moderate and high groups. Therefore, the differences between these two groups may have lied instead on participants’ abilities to overcome any barriers to adherence they may have faced. Second, because of the large gap in time between the closure of FEM-PrEP and the implementation of the follow-up study, recruiting former FEM-PrEP participants with the most specimens available and reaching our sample size (particularly in the high adherence group in Pretoria) were difficult because many participants’ contact information had changed. We therefore enrolled some participants who had only a few specimens available. Yet, a strength of our study was that we explored adherence over multiple time points for many participants rather than using drug concentration data from a single cross-sectional visit. Third, our findings may not be, and were not expected to be, representative of all FEM-PrEP participants who took the study pills given our sampling and recruitment strategies. Fourth, participants in the placebo arm did not participate in SSIs before they completed ACASI as part of this study. These participants might have responded differently if they had previously discussed adherence-related issues face-to-face with an interviewer.

In moving forward, participants’ mistrust of their partners’ ability to remain HIV-negative provides further evidence that women want and need novel HIV prevention strategies. Our study’s findings can help inform both future ARV-based HIV prevention trials and the rollout of effective ARV-based HIV prevention technologies for women. Future trials should continue to describe, during community engagement, the importance of clinical research to appeal to individuals who may be interested in participating in clinical trials for altruistic reasons or out of a general interest in finding new HIV prevention products. Counseling in such trials should explore and support personal motivations for adherence while being mindful of and offering strategies to limit preventive misconceptions. In both future trials and in the rollout of effective ARV-based HIV prevention products, counseling should explore the extent to which participants wish to involve partners in their use of novel HIV prevention products, and should continue to identify ways for participants to use tools and other approaches for integrating product use into their daily lives.
